# RaTrav: a tool for calculating mean first-passage times on biochemical networks

**DOI:** 10.1186/1752-0509-7-130

**Published:** 2013-11-21

**Authors:** Mieczyslaw Torchala, Przemyslaw Chelminiak, Michal Kurzynski, Paul A Bates

**Affiliations:** 1 Biomolecular Modelling Laboratory, Cancer Research UK London Research Institute, 44 Lincoln’s Inn Fields, London WC2A 3LY, UK; 2 Adam Mickiewicz University, Faculty of Physics, Umultowska 85, 61-614 Poznan, Poland

**Keywords:** Markov processes, Master equation, Random walk, Mean first-passage time, Stationary flux, Monte Carlo method, Hill’s method, Complex networks, Chemical kinetics, Protein-protein binding funnel, Protein-protein interactions, Protein-protein docking, Free energy transduction

## Abstract

**Background:**

The concept of mean first-passage times (MFPTs) occupies an important place in the theory of stochastic processes, with the methods of their calculation being equally important in theoretical physics, chemistry and biology. We present here a software tool designed to support computational biology studies where Markovian dynamics takes place and MFPTs between initial and single or multiple final states in network-like systems are used. Two methods are made available for which their efficiency is strongly dependent on the topology of the defined network: the combinatorial Hill technique and the Monte Carlo simulation method.

**Results:**

After a brief introduction to RaTrav, we highlight the utility of MFPT calculations by providing two examples (accompanied by Additional file 1) where they are deemed to be of importance: analysis of a protein-protein docking funnel and interpretation of the free energy transduction between two coupled enzymatic reactions controlled by the dynamics of transition between enzyme conformational states.

**Conclusions:**

RaTrav is a versatile and easy to use software tool for calculating MFPTs across biochemical networks. The user simply prepares a text file with the structure of a given network, along with some additional basic parameters such as transition probabilities, waiting probabilities (if any) and local times (weights of edges), which define explicitly the stochastic dynamics on the network. The RaTrav tool can then be applied in order to compute desired MFPTs. For the provided examples, we were able to find the favourable binding path within a protein-protein docking funnel and to calculate the degree of coupling for two chemical reactions catalysed simultaneously by the same protein enzyme. However, the list of possible applications is much wider.

## Background

The theory of stochastic Markov processes has many applications in theoretical physics, chemistry and biology [1-5]. If a system allows transitions in time between various discrete states, we may model the system in the general language of networks and assign dynamics properties to the nodes and edges of such networks [6]. In early attempts to understand the dynamics of stochastic networks the term ’random walk’ was introduced [7], which describes the displacement of a point on a network after a sequence of random moves. Equally important to measuring displacement is the quantity called mean first-passage time (MFPT), which is defined as the time needed to reach the final state by a statistical ensemble of network walkers [8]. According to the alternative definition, this time is equivalent to the reciprocal steady-state flux resulting in a network in which a single walker returns instantly to the initial state every time it reaches the final state [9].

MFPTs have been successfully used in a variety of biochemical studies, for example: the study of protein folding times [10], protein helix unfolding rates under mechanical forces [11], studies of DNA-based nanoscale walkers called molecular spiders [12], studies of polymer translocation [13], calcium spark activation times [14], metastatic cancer progression [15], and the analysis of temperature and detection-wavelength dependence of the electron transfer rates in the initial stages of photosynthesis [16].

In general, time for random walks upon a network may be discrete or continuous. The RaTrav tool works with discrete time. To our best knowledge, there is currently no open source software available which is able to perform MFPTs calculations on a discrete time and space network of any arbitrary size. In this article we present RaTrav, an open source software tool. First, we introduce the formulation of MFPT calculations and provide details on the implementation and usage of RaTrav (Methods section). Then we focus on two biological applications and demonstrate how various biological questions may be answered using the RaTrav software tool (Results and discussion section).

## Methods

### Mean first-passage times

When considering the Markov processes, MFPT is defined as the average time (in number of steps or other units of time) for a statistical sample of random walks starting in some initial state on a network of states to reach for the first time the desired final state or any state from a collection of final states. In formal terms, the stochastic continuous time Markov process realized on a given network of states is described by a system of coupled master equations

(1) $${\dot{p}}_{l}(t)\;=\;\underset{l^{\prime }}{\sum }\left[w_{ll^{\prime }}p_{l^{\prime }}(t)-w_{l^{\prime }l}p_{l}(t)\right]$$

which jointly describe the time variation of the probabilities *p*_*l*_(*t*) of the hypothetical random walker being in some state *l* at time *t*, where the over-dot denotes a time derivative. In Eq. 1, $w_{l^{\prime }l}$ is the transition probability per unit time along the edge from state (node) *l* to *l*^′^, which in general needs not satisfy the detailed balance condition. Furthermore, the transition probabilities $w_{l^{\prime }l}$ from a given state *l* to its nearest neighbours *l*^′^ need not sum up to unity. On establishing a discrete time *t* = *nτ*_0_, where *n* is the number of steps measured in some unit of time *τ*_0_, the differential Eq. 1 is to be recast as the difference master equation

(2) $$p_{l}(n+1)\;=\;p_{l}(n)+\underset{l^{\prime }}{\sum }\left[u_{ll^{\prime }}p_{l^{\prime }}(n)-u_{l^{\prime }l}p_{l}(n)\right]\;,$$

in which, as opposed to Eq. 1, the transition probabilities $u_{l^{\prime }l}\;=\;\tau_{0}w_{l^{\prime }l}$ from the node *l* to the node *l*^′^ sum up to unity:

(3) $$\underset{l^{\prime }}{\sum }u_{l^{\prime }l}\;=\;\tau_{0}\underset{l^{\prime }}{\sum }w_{l^{\prime }l}\;=\;1\;.$$

Let us note that in general the above sum includes also the waiting probabilities *u*_*ll*_ on a given node *l* before a random walker performs a jump to a neighboring node *l*^′^. They have efficient applications in many biological and physical problems. For example, a protein-protein complex residing in the bound conformational state while performing its function and before proceeding to the dissociation state, or the components of the complex becoming trapped in a local minimum of the free energy landscape before finding their optimal docked, and fully functional, conformational state. Moroever, the long-living metastable states are detected in various excited atomic or molecular systems before they relax to the lower-lying energy states.

In developing the RaTrav code we have ensured that the definitions for the input data are as general as possible. Therefore, we have made a distinction between the exit probability from a node and the transition time to its nearest neighbour. More generally, on defining the various local times $\tau_{l^{\prime }l}$, individually for each transition along a link with the probability $u_{l^{\prime }l}$, we have the alternative expression for the transition probabilities per unit time in Eq. 1:

(4) $$w_{l^{\prime }l}\;=\;\frac{u_{l^{\prime }l}}{\underset{l^{\prime }}{\sum }\tau_{l^{\prime }l}u_{l^{\prime }l}}\;.$$

In the particular case for all $\tau_{l^{\prime }l}\;=\;\tau_{0}$ we reconstruct the transition probabilities that fulfill the conditions in Eq. 3. The local times can be related to the reaction coordinates, for example, when employing molecular dynamics simulations to move between well defined protein conformational states, which might be measured on the order of microseconds.

By virtue of its generality, Eq. 4 has been directly implemented only in the Hill combinatorial technique [9]. Nevertheless, as regards Monte Carlo simulations, it ensures a determination of the same unit of time for the both methods. The description and benchmark of the combinatorial Hill and stochastic Monte Carlo methods on basic networks (equal exit probabilities towards each neighbour, equal weights of edges) may be found in our previous paper [17]. In comparison to the previous results, newly developed C++ code is provided, which allows for the definition of multiple final states, different transition probabilities and local times along network edges. Moreover, the networks can be connected or disconnected structures represented as basic graphs, as directed graphs, as multigraphs and as multi-component graphs or their mutual mixtures. In order to compare MFPTs generated by these two methods we have run a series of tests on a number of network topologies studied previously [17]: hypercubes of various dimensions, Sierpinski gaskets of various orders, Bethe lattices with various number of shells and random tree-like networks; with equal and randomly chosen probabilities, with identical and different local transition times, and with single and multiple final states. For most of the cases, the difference between MFPTs calculated from the Hill and Monte Carlo methods, calculated as 100% (|H − MC|/H), was smaller than 0.2% when using 10^7^ walkers.

Both methods mentioned above have their benefits and drawbacks. For instance, the advantage of Hill’s over the Monte Carlo method is its speed and precision of calculation when the network is an acyclic graph, or includes a low number of cycles. On the other hand, for particularly knotted networks, the Monte Carlo method is the logical choice, providing reliable MFPT estimations in a reasonable turnaround time. The best strategy to follow using the Monte Carlo method is to start with a lower number of walkers; even if the obtained results are not particularly accurate, this helps to estimate the running time with the desired higher number of walkers and avoids the situations where calculations are indicated to be intractable in a finite time. Some indication of running times may be found in Tab. 4 of [17].

### Implementation of the Monte Carlo simulation method

The Monte Carlo method relies on the simulation of random walks on a network of interest, driven by a pseudorandom number generator. Random numbers were generated in the Monte Carlo method using the Boost C++ Libraries components (http://www.boost.org) – Mersenne Twister mt19937 random number generator (with a standard seed) and a uniform_real_distribution function.

In our implementation (see source code), there is an outer for loop over the number of simulations and an inner for loop over the number of walkers in each simulation. Each walker is placed in an initial state (node on the network) and its first passage time set to 0. For each iteration, a random number is generated. Exit probabilities from each node sum up to one but do not have to be equal, thus forming a ranges of transition probabilities to each neighbour. Each transition is chosen as an effect of casting a random number. After a walker undergoes a transition it is placed in a new state (or stays in the same state if the waiting probability exists and the random walker chooses this transition) and its first passage time is increased by the weight of edge (local time) it passed. The walker performs its random walk until it reaches the final state or one state from the set of final states. When all random walkers finish their walk, the mean first-passage time is calculated based on first passage times for each walker. If the user chooses to perform more simulations with the given number of walkers, mean first-passage times are averaged further and standard deviation of this mean over the number of simulations, with the given number of walkers, is calculated. Please notice that if the number of walkers is large enough (in fact the user should perform some initial simulations to be sure the number of walkers is sufficient for a new network), MFPTs obtained from each simulation will be similar. However, because of the random movement of walkers, the first passage time of a single walker cannot be predicted based on e.g. first passage time of another walker.

In terms of efficiency of the calculations, because the current step of a walker determines its next step, a single random walk cannot be parallelized. However, the calculation of mean first-passage time may be parallelized, because the walkers in the statistical ensemble are independent. We have shown that using parallel processing methods (Message Passing Interface), the efficiency may be increased over 90% using more than 10 CPUs or by using a few cores on a single CPU [18]. However, to keep the RaTrav software as portable as possible, we haven’t implemented MPI parallel processing methods in the current version of the software.

### Implementation of the combinatorial Hill method

The Hill method relies on the idea that instead of an ensemble of walkers one can consider only a single walker that after traversing a network of states (nodes) appears instantly at the starting node every time it reaches the target node. Because this procedure is repeated many times the Hill method shows that the MFPT *τ* between these two nodes corresponds to the reciprocal one-way stationary flux *J*, *τ* = *J*^−1^, resulting in a modified network in which the transitions to the target node have been redirected to the starting node. The steady-state flux has the following form

(5) $$J\;=\;\underset{l^{\prime }}{\sum }w_{ll^{\prime }}p_{l^{\prime }}^{\text{st}}\;,$$

where $w_{ll^{\prime }}$ stands for the transition probabilities per unit time from a set of *l*^′^ nodes adjacent to the target node *l* and occupied by the walker with probabilities $p_{l^{\prime }}^{\text{st}}$. We can calculate these probabilities solving the system of the stationary master equations in Eq. 1 for ${\dot{p}}_{l}(t)\;=\;0$, or equivalently using the Hill algorithm [9] that we have now implemented in the RaTrav tool. The algorithm proceeds according to the following steps: 

1. Determine two nodes on the original network (graph) in order to calculate the MFPT between them.

2. Modify this graph through the identification of the initial node with the final node, combined with elimination of the latter.

3. Construct for such a modified graph *G* the complete set of its subgraphs, called maximal trees *T*. The maximal tree is a connected graph which contains all nodes of the graph *G* and no cycles.

4. Make each possible maximal tree *T* to be a directed graph. It is obtained from *T* by directing all its edges (links) towards the node *l*. Each directed tree *T*_*α*_ contributes a weight *W*_*l*_(*T*_*α*_) as the product of transition probabilities per unit time *w*_*ij*_ from the node *j* to the node *i*.

5. Calculate the sum of these weights, $W_{l}\;=\;\underset{\text{all}\;T_{\alpha }}{\sum }$*W*_*l*_(*T*_*α*_), which run over all maximal trees *T*_*α*_ directed to a given node *l*.

Then, the steady-state occupation probability of the node *l* in the network (graph) *G* becomes

$$p_{l}^{\text{st}}\;=\;\frac{W_{l}}{\underset{n}{\sum }W_{n}}\;,$$

where the expression in the denominator obeys a summation over all sum of weights generated for the graph *G* to ensure that $\underset{n}{\sum }p_{n}^{\text{st}}\;=\;1$. The construction of these probabilities is fundamental for the calculation of the stationary flux in Eq. 5 and finally the MFPT. To this end we have applied the algorithm which for a given set of elements (the graph edges) generates its subset in lexicographical order.

### Input file and control keywords

The user is required to prepare a text file with the structure of a given network. Upon completion of the computations, an output file is produced with computed MFPTs along with, in case of the Monte Carlo method, their estimated errors. The user needs to choose between the Hill’s and Monte Carlo methods, between using basic (just neighbouring nodes) or advanced (with transition probabilities and local times along edges), between calculating all the MFPTs or only a selection, and whether to define multiple final states.

Let us start with a very simple square network for which the input file in RaTrav format takes the following form:

Keyword NODE is followed by the node number (the first numerical column, entries are counted from 0; it is important to maintain an increasing numeric order of node identities and gaps in numbering are not permitted). Subsequent numbers are the identities of neighbouring nodes and their order does not matter. In the case of performing a Monte Carlo simulation there is the requirement for WALK and SIMU keywords (number of walkers, number of simulations with WALK walkers). Entering SIMU >1, the user will receive error estimates for each MFPT calculation (standard deviation of average MFPT over the number of simulations run). To establish waiting probabilities on, for example the 9th node, the NODE 9 input line may be defined as:

i.e. the node number is repeated. This means that the node labelled as 9 in a graph connects directly to the node labelled as 3 and to itself twice, so the transition probability from node 9 to node 3 is 1/3, whereas the waiting probability on the node 9 is 2/3. If the user chooses to use Hill’s algorithm, WALK and SIMU keywords won’t be used even if present in the input file.

In advanced file format the user can define transition probabilities and weights of edges. To minimize rounding errors, in addition to the decimal format, it is possible to define the transition probabilities and weight of edges as common fractions. The same square can be defined as follows (node number, followed by a triplet containing: identity of neighbouring node, transition probability, weight of edge):

With transition probabilities equal to 1/2 and weights of edges equal to 1.0, this format is equivalent to the basic file format. However, in advanced file format it is possible to use different combinations of probabilities and weights. Requirements are that the probabilities in each line have to sum up to 1.0, and in the case of Hill’s method, at least one local timescale (weight of edge) must be different from 0. The last condition does not apply to the Monte Carlo method. Thus, on account of some specific problem, the advanced input file may take the following form:

In the above please note the different values of weights possible for two-way transitions between the same pair of nodes, for example, 1 and 3. We assume in general that a passage in either direction along a given edge does not need to correspond to the same weight or transition probability.

Optional use of the MFPT keyword allows definition of selected MFPTs the user wants to be computed, e.g.: MFPT 0 1 means the MFPT between states 0 and 1 will be calculated, MFPT 0 1 2 means the MFPT between initial state 0 and two final states will be calculated – either state 1 or 2 has to be reached (it is possible to define any number of final states).

The INFO keyword allows the user to pass any comments which will be copied to the output files.

### Compilation and usage

There is a Makefile attached so under Linux it is sufficient to simply call make. However, two variables have to be set before doing so:

The first variable is used to choose the compiler, e.g. icc for Intel C Compiler or g++ for GNU C Compiler. The Monte Carlo method uses Boost C++ Libraries (http://www.boost.org) which have to be downloaded and installed. The second variable is used to set the path to the Boost C++ Libraries so the user must change PATH_TO_BOOST to the local path. Alternatively the RaTrav tool may be compiled without the Makefile, e.g. with ICC, as follows:

The general usage is as follows:

The five parameters have the following meaning: input is a name of the input text file which defines the network; output is a name of the output text file generated by RaTrav; the METHOD parameter that should be set equal to 0 to use the Monte Carlo method or 1 for Hill’s method; the INPUT_FORMAT parameter should be set equal to 0 for a basic input file or 1 for an advanced input file; the MODE parameter should be set equal to 0 when all MPFTs are to be calculated or 1 when selected MFPTs are to be calculated (MFPT keyword needed as introduced above).

For example: to use the Monte Carlo method, with a basic input file and for all MFPTs to be calculated, the user runs RaTrav input.txt output.txt 0 0 0; to use Hill’s method, with an advanced input file and for selected MFPTs to be calculated, the user runs RaTrav input.txt output.txt 1 1 1.

### A basic example

**Figure 1:**
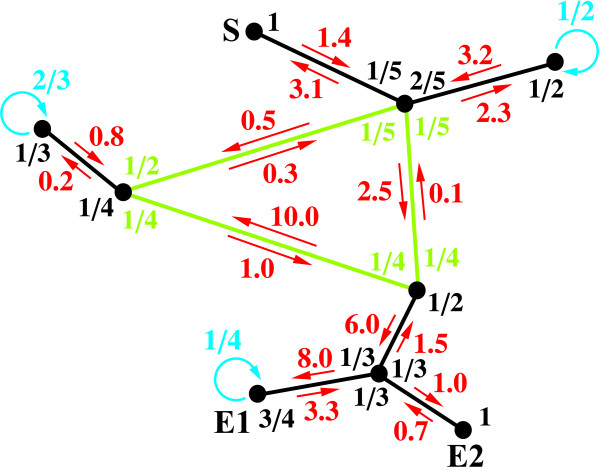
**Dynamics on a network.** A simple network with nine nodes and its properties (transition probabilities (black), local times scales (red), cycle (green), probability of waiting (cyan), initial (S) and final (E1, E2) states). See text for more details.

To better explain the functionalities of the RaTrav tool, we present in Figure 1 a simple irregular network with nine nodes (black dots) and nine edges (links between nodes). It has one cycle (edges coloured in green); nodes have different exit probabilities $u_{ll^{\prime }}$ (probabilities of passing defined as common fractions; coloured in black, in case of a cycle in green, in case of waiting on a node in cyan), and local time scales $\tau_{ll^{\prime }}$ (the cost of passing defined as decimal fractions with arrows; coloured in red). For some nodes there are waiting probabilities (i.e. transitions to the same node; cyan circular arrows); sometimes the transition probability from an edge node is set equal to one, instead of a waiting probability with an associated waiting time (weight of self transition). Both variables $u_{ll^{\prime }}$ and $\tau_{ll^{\prime }}$ may be symmetric or asymmetric. The weight of an edge is the time required for transition between states, whereas the waiting time is the lifetime of the molecule in a particular state. These times can be related to experimental values, for example, the half-life of a complex can be calculated from its dissociation rate *k*_off_, *τ*_1/2_= ln(2)/*k*_off_[19].

The network presented in Figure 1 (input and output files may be found in Additional file 1) is represented in RaTrav format as follows (nodes are numbered from the left to the right, and from top to bottom, so S=0, E1=7, E2=8):

Please note that node numbers must be written in increasing order, but the neighbours of each node may be written in any order. If we add the following lines to the input file:

the output using Hill’s method (WALK and SIMU not used) is:

and with the Monte Carlo method (WALK and SIMU used) is:

For the presented network, the MFPT between S and E1 is calculated to be 80.300, between S and E2 to be 83.233, and between S and E1 or E2 (multiple final state) to be 61.556. Two observations can be made: for the submitted network Hill’s method is faster and gives exact results; using the Monte Carlo method, with 1000000 walkers, gives similar results. In case of reducing the number of walkers to 100000 but increasing the number of simulations to 10:

the following results with Monte Carlo can be obtained:

In this case the program also returns the standard deviation for each MFPT; comparing each MFPT, and its associated error, with the equivalent but exact result from Hill’s method, we may notice that one standard deviation may be insufficient to denote each MFPT pair to match; however, within three standard deviations each MFPT pair should match. This may not always be the case for more complicated networks, since if the number of walkers in each simulation is too low, every node may not be visited by a walker from the ensemble (ergodicity issues).

### Important remarks

1. When using the Hill method the final state must be different from the initial state. However, when using the Monte Carlo method one can identify such states and obtain the MFPT, which is in fact a return time to the origin.

2. For the Hill method the number of final states cannot exceed N-2, where N is the total number of states (nodes in a graph). However, for the Monte Carlo method one can define such a configuration of states, e.g. MFPT 0 1 2 3 for a square.

3. By choosing the Monte Carlo method the user is able to define ultrafast transitions between all or selected states, for which the local passage time along an edge between such states is set equal to zero. For the Hill method at least one local time scale must be different from zero.

4. A network does not have to be compact. If there is no path between states, RaTrav will return MFPT as ’Infinity (not accessible)’.

## Results and discussion

In this section we provide two applications for which the use of the RaTrav tool dedicated to MFPT calculations provides meaningful results. Each subsection includes a theoretical introduction to the problem, followed by a guide for the reader as to how to construct the appropriate RaTrav input files, and finally a discussion of the RaTrav results. The files accompanying the examples are available in Additional file 1.

### Analysis of conformational pathways within a protein-protein binding funnel

In the molecular machinery of life proteins are responsible for a diverse array of functions. However, the great majority of biological functions are mediated not by isolated proteins but by their interactions. In addition to predicting the correct geometry of protein-protein complexes (in 3D) from their unbound components, for which a number of fully automated servers now exist, see for example the SwarmDock server [20] or the ClusPro server [21], of equal importance is to study the dynamics of binding, i.e. how the binding partners, upon complex formation, sample the binding funnel.

Studying the topological properties of protein-protein binding funnels will enable us to understand how to change the dynamics of protein-protein association in a controlled way. The importance of being able to do this relates to rational drug design, where funnel sampling becomes particularly important when designing a series of similar protein ligands (such as proteins with a few key point mutations), or blocking peptides, and ascertaining if they are likely to be more effective in inhibiting a particular receptor protein-binding site more than the wild-type protein ligand.

Automatic generation of protein-protein conformational space networks (in RaTrav formatted files), for any protein receptor/ligand pair, was recently incorporated into our docking tool, the SwarmDock Server [20]. For this study, we chose the vitamin D-binding protein/actin complex (Brookhaven protein database code, 1KXP) [22], which was previously studied by us in terms of conformational occupation probabilities and their usefulness to filter away non-funnel like protein-protein energy structures, thus improving the ranking of the correct docking poses [23]. The above study, based on state occupancies, was useful in distinguishing between true positive and false positive binding funnels. In the example described below we focus on the properties of the true positive binding funnel and calculate mean first-passage times between distinct conformational states within the funnel, i.e. we are interested in finding the favourable transition path from the top to the bottom of the binding funnel.

**Figure 2:**
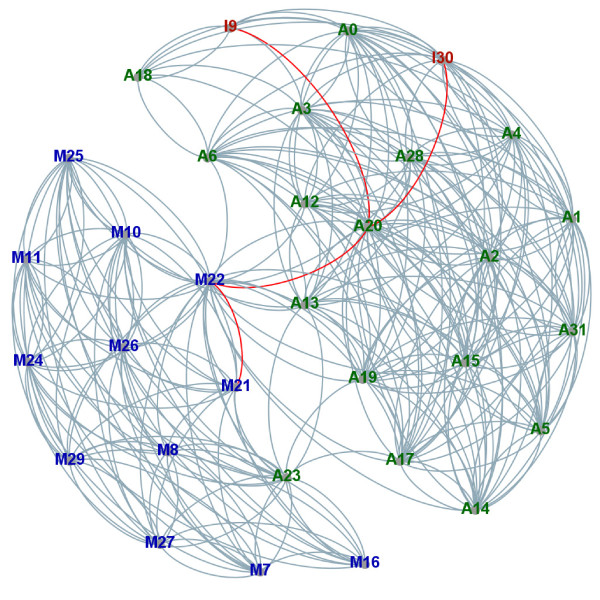
**The true positive binding funnel.** The true positive binding funnel for the vitamin D-binding protein/actin complex (1KXP [22]) generated with the SwarmDock Server [20] by docking unbound receptor/ligand pair included in Benchmark 4.0 [25]. The protein-protein conformational states are numbered from ID 0 to 31. The letter indicates the quality of solution in accordance to CAPRI [24] classification as: M – medium quality (blue), A – acceptable quality (green), I – incorrect solution (red). Favourable paths are marked in red. Figure created with Gephi [26] based on Example1/1KXP.gml file (Additional file 1). See text for more details.

The initial network of 32 conformational states, generated by the SwarmDock server, is depicted in Figure 2. The assigned quality of each state, that is its similarity with the final bound complex state, was based in accordance to the CAPRI (Critical Assessment of PRediction of Interactions) criteria [24], on three quantities: fraction of native contacts (fnat), interface root mean square deviation (IRMSD) and ligand root mean square deviation (LRMSD). These values are used to classify the conformations as incorrect (fnat < 0.1 or (LRMSD > 10Å and IRMSD > 4Å)), acceptable ((fnat ≥ 0.3 and LRMSD > 5Å nd IRMSD > 2Å) or ((fnat ≥ 0.1 and fnat < 0.3) and (LRMSD ≤ 10Åor IRMSD ≤ 4Å))), medium quality ((fnat ≥ 0.5 and LRMSD > 1Å and IRMSD > 1Å) or ((fnat ≥ 0.3 and fnat < 0.5) and (LRMSD ≤ 5Å or IRMSD ≤ 2Å))) or high quality (fnat ≥ 0.5 and (LRMSD ≤ 1Å or IRMSD ≤ 1Å)), relative to the conformation of the native bound complex (i.e. the conformation at the bottom of the binding funnel).

Similarly to the previous study [23], the transition probability for states with ligand RMSD above 6Å was set to zero. For the remaining states, transition probabilities were assigned based on energy value (the OPUS-PSP potential [27]): if the energy of the state in question was higher than the neighbouring state, the exit probability was set to 1.0, otherwise it was set to exp(−*ΔE*), where *ΔE* is the difference in the energy between the two conformations. The exit probabilities for each node were normalized. In the present study, the weights of each edges are set equal to 1.0 (global time scale, we assume that conformational states are sampled uniformly in time), and if the transition probability is smaller than 10^−6^ for a particular edge it is removed. PDB files (Example1/*.pdb), the RMSD matrix file (Example1/1KXP.rmsd), the MFPT matrix file (Example1/1KXP.mfpt) and characteristics of the conformational states file (Example1/1KXP.info) are deposited in Additional file 1. Here we are interested in finding the favourable path(s) between incorrect conformational states (ID 9 and 30; states on the edge of the binding funnel) and the best state found by SwarmDock (the state closest to the bottom of the binding funnel; the native complex state). The state identified as being the closest to the native complex state, based upon the CAPRI criteria described above, is M21 (see Figure 2 and Example1/1KXP.info for details).

The RaTrav tool was run on this network (Example1/1KXP.ratrav) with 10^6^ random walkers. To speed up the computations we parallelized the calculations by creating a separate input file for each pair of states and by using the MFPT keyword (see Methods). We ran the calculations on our computational cluster (HP ProLiant BL460c) in parallel for 992 MFPTs (32∗32−32) for a maximum walltime of 14 days. A total of 291 MFPTs were reported to be not accessible; links between nodes with transition probabilities smaller than 10^−6^. Of the remaining 701 MFPTs a total of 372 were reported back by RaTrav, the remainder, 329 were still in the process of being computed and assumed to be essentially infinite; that is a substantial number of the network walkers were stuck in dead ends.

Using the initial network (Example1/1KXP.ratrav), we assigned weights to edges based on calculated MFPTs; we removed links if MFPTs haven’t been calculated. On this MFPT weighted network (all weights are positive) we ran the Dijkstra algorithm [28] to find the favourable trajectories (shortest paths in units of mean first-passage times) between conformational states on the edge of the binding funnel (I9 and I30) and the medium quality structure near the bottom of the funnel (M21).

The Dijkstra algorithm identified the following shortest paths: 9→20→22→21 (sum of MFPTs equals 4,624,065 steps) and 30→20→22→21 (sum of MFPTs equals 4,243,494 steps).

We analyzed the trajectory in terms of changes in interface and ligand RMSD, number of native and non-native contacts and energy of each conformational state. We summarize the calculations in Table 1 and the values for all states are present in Additional file 1 (Example1/1KXP.info).

**Table 1:** Conformational states in the favourable trajectory from the edge of the binding funnel I9 or I30 to M21 near the bottom of the funnel

**Node ID** **(Figure **2**)**	**IRMSD**	**LRMSD**	**fnat**	**fnonnat**	**OPUS-PSP** [27]
9 (59b.pdb)	4.41	15.43	0.22	0.78	-335.113
or					
30 (99c.pdb)	4.01	14.81	0.25	0.77	-385.907
20 (79a.pdb)	2.95	10.60	0.42	0.60	-441.811
22 (80c.pdb)	1.88	5.53	0.61	0.42	-405.242
21 (80a.pdb)	1.41	3.14	0.67	0.31	-406.366

Interface RMSD (IRMSD), ligand RMSD (LRMSD), number of native contacts (fnat), number of non-native contacts (fnonnat) and OPUSPSP energy values are reported. The first four values were computed comparing with the native complex structure.

The final state is accessed slightly faster from the edge state I30 which has fnat = 25% than the edge state I9 with the slightly lower fnat = 22%. The difference in paths is only due to the MFPT for the first transition, to the state A20. Interestingly, the initial transitions (IDs 9→20 or 30→20) are down an energy gradient, at least in terms of the OPUS-PSP potential used to score protein-protein interactions. However, the next two transitions, A20→M22→M21 are movements to slightly increased energy states, indicating that the most time efficient pathways do not necessarily follow a decreasing energy gradient. Moreover, in terms of MFPTs, the transition between states A20 and M22, which looks in Figure 2 to be a bottleneck (kinetic trap), is not the limiting transition when comparing MFPTs for the first transition (I9 or I30 to A20) or the last transition in the pathway (M22 to M21).

In conclusion, by exploring MFPTs between conformational states within a protein-protein binding funnel, dynamic information can be obtained that may provide important complementary, and sometimes counter intuitive, information on the funnel’s physical properties, which may facilitate rational design of competitive protein ligand inhibitors.

### Free energy transduction between two coupled enzymatic reactions

As a second example of the utility of RaTrav, we describe here a method that enables a user to determine the non-equilibrium stationary fluxes in a system of interest on the basis of MFPT calculations. To this end we consider the action of a protein enzyme that converts the free energy between two coupled chemical reactions [29]**,**[30]. Our primary task is to calculate the degree of coupling between the free energy-donating reaction and the free energy-accepting one, the parameter that characterizes the efficacy of this chemo-chemical machine.

**Figure 3:**
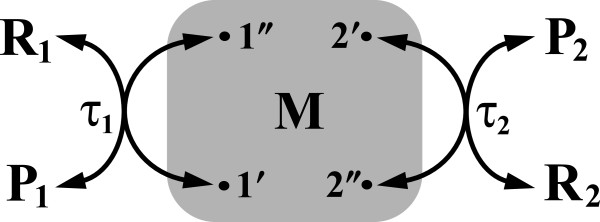
**Chemo-chemical molecular machine.** A scheme of two coupled free energy-donating R_1_** ⇔ **P_1_ and free energy-accepting R_2_** ⇔ **P_2_ enzymatic reactions involving the intramolecular conformational dynamics of the enzyme macromolecule. The multitude of conformational transitions within the complex M is represented by the shaded box. Both coupled reactions are assumed to be gated by certain conformational substates distinguished here as the black dots. The transitions between gates are characterized by the external transition times *τ*_1_ and *τ*_2_.

**Figure 4:**
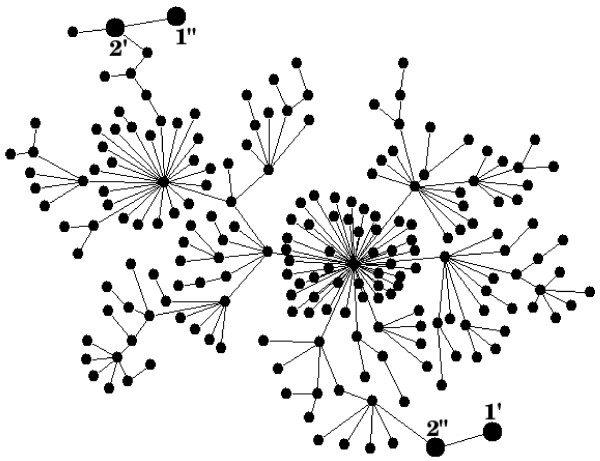
**The model network of conformational substates.** The model network of stochastic transitions between conformational substates (nodes) corresponding to the shaded box M in Figure 3. Its structure displays both the scale-free topology, as well as the fractality [31]. The distinguished nodes (enlarged dots) symbolizes the gates which have been marked here in accordance with a system of labels used in Figure 3.

Let us consider the shaded box shown in Figure 3 which represents the network of conformational substates of a protein macromolecule that catalyses simultaneously two, in general, reversible reactions: the free energy-donating reaction R_1_** ⇔ **P_1_ and the free energy-accepting reaction R_2_** ⇔ **P_2_. A model structure of such a network consisting of two hundred nodes is depicted in Figure 4. A set of distinguished transition states, called the gates, underscored by enlarged black nodes, corresponds directly to the system of labels used in Figure 3. For the sake of clarity, we have limited our calculations to a rather simple network of states displaying a tree-like topology, but much more complex networks of states can also be taken into account. In this context, the actual network can be thought of as a spanning tree, a loopless subnetwork consisting of edges with the highest transition probabilities per unit time between conformational substates.

The steady-state reaction fluxes

(6) $$J_{i}\equiv \;[\;{\dot{P}}_{i}]/{\left[\;E\right]}_{0}$$

(*i* = 1,2) determine by definition the effective reaction rates, where [ E]_**0**_ is the total concentration of the enzyme molecule and the over-dot means a derivative with respect to time. The corresponding chemical forces

(7) $$\beta A_{i}=\ln K_{i}\frac{\left[\;R_{i}\right]}{\left[\;P_{i}\right]}\;,\;\;\;\;\;K_{i}\equiv \frac{{\left[\;P_{i}\right]}^{\text{eq}}}{{\left[\;R_{i}\right]}^{\text{eq}}}\;,$$

drive both chemical reactions. The symbols enclosed in the square brackets denote the molar concentrations of the chemical compounds R_*i*_ and P_*i*_ in the steady state (no subscript) or in the equilibrium state (the superscript eq). Here, *β* = (*k*_**B**_*T*)^**−1**^, where *k*_**B**_ is the Boltzmann constant and *T* denotes the absolute temperature.

The protein enzyme acts as a mesoscopic molecular machine, i.e. a system that enables two subsystems to perform work on one another, with the efficiency given by the ratio

(8) $$\eta \;=\;-J_{2}A_{2}/J_{1}A_{1}\;,$$

of the output power *J*_2_*A*_2_ to the input power *J*_1_*A*_1_. The minus sign in Eq. 8 arises from the fact that the free energy transduction can be realized only if the positive flux *J*_2_ > 0 corresponds to the negative chemical force *A*_2_ < 0 or vice versa [9]. This contradicts the second law of thermodynamics according to which the flux as well as the force should be of the same sign. However, if both reactions are coupled by the protein enzyme and proceed simultaneously in a common cycle, then the first reaction can change the direction of the second reaction. Consequently, the free energy dissipation is minimized at the expense of the free energy conversion, which can be realised with higher efficacy in the system. The effectiveness of this process is characterised by a degree of coupling

(9) $$\varepsilon \;=\;J_{2}/J_{1}\;.$$

In the following, our main objective is to give some instructive hints of how to calculate a degree of coupling for the two chemical reactions controlled and gated by the network of conformational transitions depicted in Figure 4. This network forms the content of the grey box, representing the enzyme-substrate(s) complex M, shown schematically in Figure 3. We assume that the stochastic dynamics of conformational and chemical transitions within the enzymatic protein is jointly described by the system of master equations (see Eqs. 1 or 2). For the isolated network (without the chemical reactions) we assume the transition probabilities per unit time from the conformational substate *l* to its *k*_*l*_ directly adjacent substates *l*^**′**^ to be

(10) $$w_{l^{\prime }l}\;=\;p/k_{l}.$$

*p* determines the probability of transition to any conformational state neighbouring *l*. Following the detailed balance principle

(11) $$w_{ll^{\prime }}p_{l^{\prime }}^{\text{eq}}\;=\;w_{l^{\prime }l}p_{l}^{\text{eq}}$$

the equilibrium occupation probability of the substate (node) *l*

(12) $$p_{l}^{\text{eq}}\;=\;\frac{k_{l}}{\underset{l^{\prime }}{\sum }k_{l^{\prime }}}\;,$$

where the summation runs over all nodes composing a network of conformational substates.

The non-zero thermodynamic forces, Eqs. 7, drive the system out of equilibrium breaking simultaneously the detailed balance condition for external and in general reversible transitions between gates (the transition states), which for *l* = 1,2 are characterised by the external transition times *τ*_*l*_ (cf. Figure 3). These parameters can be compared to the longest MFPT within a network of states (nodes), which usually refers to the passage time, counted in the random walker steps, between the most distant pair of nodes. Together with the equilibrium occupation probabilities in Eq. 12 they determine the additional transition probabilities between the gates in the forward direction (exit from the gate *l*^**′′**^)

(13) $$v_{+l}\;=\;\frac{p}{\tau_{l}p_{l^{\prime \prime }}^{\text{eq}}}\;,$$

and in the backward direction (exit from the gate *l*^**′**^)

(14) $$v_{-l}\;=\;\frac{p\;e^{-\beta A_{l}}}{\tau_{l}p_{l^{\prime }}^{\text{eq}}}\;.$$

The factor $e^{-\beta A_{l}}$ breaks the detailed balance symmetry. Here, the index *l* = 1,2 and the appropriate selection of primes and double primes is explained in Figure 4.

It follows from Eq. 13 and 14 that the probability *p* we introduced in Eq. 10 determines the characteristic unit of time which establishes an elementary time scale for the computer machine step. Thus, it should be firmly emphasized that the adequate determination of this quantity has a decisive meaning for the computational purpose at hand. To find *p* we select one gate *l* for which the external transition probability *v*_*nm*_ is the highest. Then, assuming that at this gate the sum *p* + *v*_*nm*_ = 1, we obtain the appropriate expression for *p*:

(15) $$p\;=\;\frac{1}{1+{\left(\tau_{l}\;p_{l}^{\text{eq}}\right)}^{-1}}\;.$$

Consequently, in the case of the remaining nodes the sum of internal and possibly external transition probabilities must be complemented to unity, which means that additionally the non-zero waiting probabilities have to be taken into account on these states.

For the network of states depicted in Figure 4 (Example2/ratrav_fig4.in in Additional file 1) the longest MFPT, *τ*_**max**_, counted in random walk steps without external transitions, equals 3261 (see Example2/HI_ratrav_fig4.out in Additional file 1); the transition state with the lowest occupation probability corresponds to the gate *l* = 1^**′′**^ (although *l* = 1^**′**^ is another good choice), for which $p_{1^{\prime \prime }}^{\text{eq}}\;=\;1/398$ (see Eq. 12 with $k_{1^{\prime \prime }}\;=\;1$, $\underset{l}{\sum }k_{l}\;=\;2\;(N-1)$ where *N* = 200 is the number of nodes in the network). Without loss of generality we consider in what follows only the case for *τ*_**1**_ = *τ*_2_. Selecting *τ*_1_ = 40, which is far below the maximal value *τ*_**max**_ = 3261, we enforce the chemical reactions to be completely controlled by the internal dynamics of conformational transitions for which *p* ≈ 0.091 in Eq. 15. Moreover, assuming *βA*_1_ = 10 we require that the free energy-donating reaction proceeds sufficiently far from equilibrium in the forward direction 1^**′′**^** → 1**^**′**^, while becoming almost negligible in the backward direction 1^**′**^** → 1**^**′′**^. In turn, we put *βA*_2_ = 0 to provide the detailed balance symmetry for the free energy-accepting reaction. Such a choice of parameters *τ*_*l*_ and *A*_*l*_ for *l* = 1,2 assures the highest transition probability from the node *l* = 1^**′′**^.

**Figure 5:**
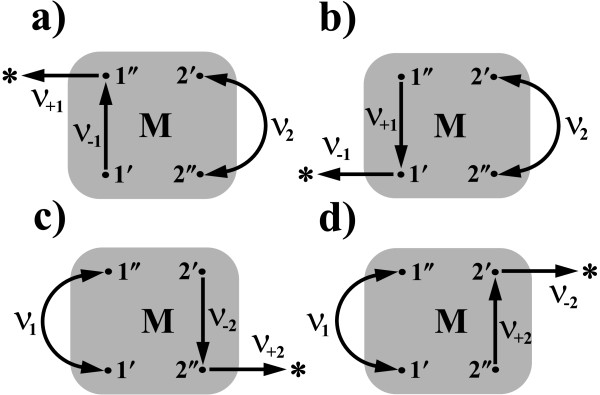
**Stationary reaction fluxes.** Four modified versions of the network of conformational substates with the internal and external transitions between them pictured in Figure 3. The considered schematic diagrams enable calculation, by using the Hill or Monte Carlo methods, the steady-state fluxes for the input/output reactions in the forward **(a)**/**(c)** and the backward **(b)**/**(d)** directions. The successive external transitions have been redirected to the additional node connected to the network and labeled here by the symbol, ∗. For instance, the forward reaction flux **(a)** is equivalent to the reciprocal MFPT from the gate 1^**′**^ to the node ∗, through the intermediate gate 1^**′′**^.

We are now in a position to determine the steady-state reaction fluxes for both chemical transitions. The net fluxes are decomposed into one-way fluxes

(16) $$\begin{array}{ll} J_{1}\; & =\;J_{+1}\;-\;J_{-1}\; \end{array}$$

(17) $$\begin{array}{ll} J_{2}\; & =\;J_{+2}\;-\;J_{-2}\;,\; \end{array}$$

where the signs plus and minus refer to the forward and the backward directions, respectively. To find them we must perform four independent calculations. Following the Hill reasoning [9] described in Methods, instead of the one-way steady state fluxes, it is enough to calculate the MFPTs between correctly identified nodes in the modified network of states. The schemes illustrated in Figure 5, explain in a pictorial form the direct relation between the MFPT and a one-way stationary flux *J*_±*l*_. According to the Hill algorithm

(18) $$\begin{array}{ll} J_{+l}\; & =\;\frac{1}{\tau (l^{\prime }\to *)}\; \end{array}$$

(19) $$\begin{array}{ll} J_{-l}\; & =\;\frac{1}{\tau (l^{\prime \prime }\to *)}\;,\; \end{array}$$

where ∗ is the additional target node connected to the network in a position where the intermediate nodes (gates) *l*^**′′**^ for *J*_+*l*_, or *l*^**′**^ for *J*_−*l*_ are localised (see Figure 5). The algorithm allowing a calculation of the one-way stationary flux between two directly connected states (nodes) in the arbitrary network of states proceeds in the following steps: 

1. Create a network (graph) with connections (links) between nodes in accordance with the RaTrav input file format (see input files in Example2).

2. Select two nodes connected directly by the link. In Figure 5a it is the reversible external transition between the node 1^**′**^ and the node 1^**′′**^ characterized by the exit probabilities *v*_**±1**_.

3. Choose a direction in which the flux will be calculated. In Figure 5a the flux *J*_+1_ is assumed to be in the counterclockwise direction.

4. Redirect one of the two (if reversible) transitions along a link to the additional node added to the original network. In Figure 5a the transition from the node 1^**′′**^ to the node 1^**′**^ has been redirected to the new node labeled there by the star.

5. Calculate the MFPT between the initial node (1^**′**^) and the final node (∗).

6. The inverse of this MFPT determines the one-way stationary flux.

**Figure 6:**
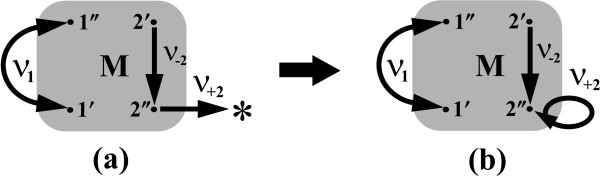
**Conformational dynamics and chemical kinetics.** Calculation of the MFPT from the node 2^**′**^ through 2^**′′**^ to ∗ on the network shown in Figure 5c can be performed using Eqs. 20 and 21as follows. First, it is sufficient to modify the initial network **(a)** by replacing the directed link from the node 2^**′′**^ to the node ∗ with the link redirecteded to the node 2^**′′**^. Then, we calculate in a such prepared network **(b)** the MFPT from the node 2^**′**^ to the node 2^**′′**^ using Monte Carlo or Hill’s method and simultaneously the steady-state occupation probability $p_{2^{\prime \prime }}^{\text{st}}$ of node 2^**′′**^, which we next multiply by the transition probability $v_{+2^{\prime \prime }}$ to ∗. The inverse of this product plus the MFPT between nodes 2^**′**^ and 2^**′′**^, sum up to the total MFPT obtained for the original network **(a)**.

Interestingly, the MFPTs in Eqs. 18 and 19 turn out to be split into two parts

(20) $$\begin{array}{ll} \tau (l^{\prime }\to *)\; & =\;\tau (l^{\prime }\to l^{\prime \prime })\;+\;{\left(v_{+l}\;p_{l^{\prime \prime }}^{\text{st}}\right)}^{-1}\; \end{array}$$

(21) $$\begin{array}{ll} \tau (l^{\prime \prime }\to *)\; & =\;\tau (l^{\prime \prime }\to l^{\prime })\;+\;{\left(v_{-l}\;p_{l^{\prime }}^{\text{st}}\right)}^{-1}\;.\; \end{array}$$

(see Theorem 2 in [30]). Here, the superscript ‘st’ indicates the stationary occupation probabilities of the selected gates which can be also computed by the RaTrav tool. A method of how to calculate all quantities, for example, on the r.h.s of Eq. 20 for *l* = 2 is explained graphically in Figure 6. The relations in Eqs. 20 and 21 offer a very useful way in which all component MFPTs from states *l*^**′**^ or *l*^**′′**^ to ∗ and hence the stationary fluxes in Eqs. 18 and 19 can be calculated. Moreover, they are of particular importance since they enable us to identify scales of two characteristic time contributions, distinguishing the conformational dynamics within the protein enzyme (the first component) and between two coupled chemical reactions each of which is gated by a single transition substate (the second component). In this second case both components, $v_{\pm l}p_{\pm l}^{\text{st}}$, can be thought of as the counterparts of the reciprocal equilibrium rate constants supposed by the transition state theory.

Input data used to compute all directed reaction fluxes in the network of states shown in Figure 4, accompanied by the outputs, are to be found in Additional file 1. The MFPTs required for a determination of the steady-state fluxes in Eqs. 18 and 19 and obtained by using of the Hill algorithm are as follows: *τ*(1^**′**^→∗) = 5050.17, *τ*(1^**′′**^→∗) = 5086078.41, *τ*(2^**′**^→∗) = 423.29 and *τ*(2^**′′**^→∗) = 461.62, whereas those obtained by application of the Monte Carlo simulations for WALK=1000000 and SIMU=1 are: *τ*(1^**′**^→∗) = 5048.95, *τ*(1^**′′**^→∗) = 5081053.61, *τ*(2^**′**^→∗) = 425.39 and *τ*(2^**′′**^→∗) = 463.55. Therefore, combining these numerical results, we conclude from Eqs. 16, 17, 18, 19 and 9, that the degree of coupling is

() $$\begin{array}{lll} \varepsilon \; & =\;0.992,\;\;\text{following the Hill method, and}\; & \;\\ \varepsilon \; & \approx \;0.978,\;\;\text{following the Monte Carlo method.}\; & \; \end{array}$$

It is worth emphasizing that both numbers are very close to unity and this result requires sensible interpretation. Let us recall that the degree of coupling is defined as the ratio of the free energy-accepting reaction flux and the free energy-donating reaction flux. We have assumed that both reactions are controlled (*τ* = 40 ≪ *τ*_**max**_ = 3261) and simultaneously gated by the dynamics of stochastic transitions within a network of conformational states. The system of connections in this network is not arbitrary but displays the scale-free topology and fractality. The fractality results mainly from the effective repulsion between the most connected nodes, so called hubs, which tend to link to small degree nodes and not to each other. Two main hubs are clearly visible in Figure 4. Their existence enables the most distant separation between the pairs of gates (transition states) for the output and input reactions. As a consequence, the MFPTs between the gates shown in Figure 4 become comparable and hence the degree of coupling is very close to unity. We have examined that all other configurations of the gates decreases the value of this parameter. There is still an open question for scientists of how to construct the effective networks of states for the real-world protein machines to predict and explain their basic functions. We hope, at least to some extent, that our software contributes to manage this task.

## Conclusions

There is a wide range of problems related to the theory of stochastic processes where the mean first-passage time quantity may be applied to describe the dynamics of the networked system. RaTrav is a software tool for calculating MFPTs on any arbitrary network or graph representing a substrate for Markovian processes, defined by the users in accordance with their requirements. MFPTs may be calculated between a pair of states or between an initial state and multiple final states. Moreover, exit probabilities from the nodes as well as local time scales along the edges may be assigned by the user. A choice between two MFPT calculation methods is made available: a stochastic Monte Carlo method and the combinatorial Hill method. To highlight the usefulness of the RaTrav tool, we presented in this article two examples of biochemical processes where the calculation of MFPTs plays an important role. For the first example (analysis of a protein-protein binding funnel), due to the large number of cycles within the network, the Monte Carlo method was applied. For the second, a tree-like network of conformational states for an enzyme, the Hill algorithm was applied in order to return results much faster and without any approximations. To our best knowledge, RaTrav is not only the first open source computational package for computing MFPTs, but as well the first computational tool to be made available where the local transition times of the network edges have been successfully introduced into Hill’s method.

We are aware that there is a substantial gap between physics and biology in terms of describing the network properties such as the proper sampling of states, assignment of transition probabilities and assignment of weights of edges (local times) in line with a chosen reaction coordinate. However, with further development in various fields, we believe that RaTrav, as a general tool, can be applied to a wide group of problems, where a biosystem or a process can be represented as a complex network.

## Availability and requirements

**Project name:** RaTrav**Project home page:** http://sourceforge.net/projects/ratrav**Operating system(s):** Platform independent**Programming language:** C++**Other requirements:** Boost C++ Libraries (http://www.boost.org/)**License:** GNU General Public License, version 3 (http://opensource.org/licenses/GPL-3.0) for RaTrav. Boost Software License (http://www.boost.org/users/license.html) for Boost C++ Libraries.

The package contains full source code, binary version of RaTrav and manual.

## Competing interests

The authors declare that they have no conflict of interest.

## Authors’ contributions

MT and PC designed, wrote and tested the RaTrav C++ code. MT and PC prepared the examples and generated the data. MT, PC and PB wrote the manuscript. All authors discussed the results and approved the final version of the manuscript.

## Authors’ information

The authors wish it to be known that, in their opinion, the first two authors should be regarded as joint first authors.

## Supplementary Material

Additional file 1 **RaTrav examples file.** File containing RaTrav input and output data for the two case studies presented. Click here for file
